# Italian translation and cultural adaptation of the communication assessment tool in an outpatient surgical clinic

**DOI:** 10.1186/s12913-016-1411-9

**Published:** 2016-04-29

**Authors:** Daniela Scala, Enrica Menditto, Mariano Fortunato Armellino, Francesco Manguso, Valeria Marina Monetti, Valentina Orlando, Antonio Antonino, Gregory Makoul, Maurizio De Palma

**Affiliations:** Medicina Nucleare AORN Cardarelli, Via A. Cardarelli , 9, 80131 Naples, NA Italy; CIRFF, Center of Pharmacoeconomics, University of Naples Federico II, Via Domenico Montesano, 49 80131 Naples, Italy; Dipartimento Chirurgico Generale e Polispecialistico, Chirurgia 3, AORN Cardarelli, Naples, Italy; UOSC di Gastroenterologia, AORN Cardarelli, Naples, Italy; Dipartimento Chirurgico Generale e Polispecialistico, Chirurgia 2, AORN Cardarelli,, Naples, Italy; Connecticut Institute for Primary Care Innovation, Hartford, CT USA; University of Connecticut School of Medicine, Farmington, CT USA; PatientWisdom, New Haven, CT USA

**Keywords:** Translation, Adaptation, Communication, Patient-physician relationship

## Abstract

**Background:**

The aim of the study is to translate and cross-culturally adapt, for use in the Italian context, the Communication Assessment Tool (CAT) developed by Makoul and colleagues.

**Methods:**

The study was performed in the out-patient clinic of the Surgical Department of Cardarelli Hospital in Naples, Italy. It involved a systematic, standardized, multi-step process adhering to internationally accepted and recommended guidelines. Corrections and adjustments to the translation addressed both linguistic factors and cultural components.

**Results:**

The CAT was translated into Italian by two independent Italian mother-tongue translators. The consensus version was then back-translated by an English mother-tongue translator. This translation process was followed by a consensus meeting between the authors of translation and investigators, and then by two comprehension tests on a total of 65 patients.

**Conclusions:**

Results of the translation and cross-cultural adaptation were satisfactory and indicate that the Italian translation of the CAT can be used with confidence in the Italian context.

**Electronic supplementary material:**

The online version of this article (doi:10.1186/s12913-016-1411-9) contains supplementary material, which is available to authorized users.

## Background

The quality of care a patient receives depends in part on the physician’s communication skills, and patient surveys consistently find that patients want better communication from their physicians. Physicians who are informative, show support and respect for the patient, and facilitate patient participation in care generally have patients who are more satisfied, more committed to treatment regimens, and who experience better health following the consultation [1–5].

Interpersonal skills are central to basic communication skills, and include the following essential elements: (1) respect, including treating others as one would want to be treated; (2) paying attention to the patient with open verbal, nonverbal, and intuitive communication channels; (3) being personally present in the moment with the patient, mindful of the importance of the relationship; and (4) having a caring intent, not only to relieve suffering but also to be curious and interested in the patient’s ideas, values, and concerns [6, 7].

Communication between surgeons and their patients is particularly important. Patients visiting surgeons are often fearful as they have to make decisions about whether to undergo invasive, often risky, procedures. The nature of these decisions is complicated and patients often lack information about surgical procedures, the options related to non-operative treatment and the requirements for rehabilitation post-surgery. Surgeons need to conduct conversation about complicated medical issues, treatment choices, complexities of surgical procedures and options, and they have to allay patients’ fears and build trust during short visits [8].

As there are currently no validated clinical scales in the Italian language to assess interpersonal and communication skills, the aim of the current study is to use a disciplined process to translate and cross-culturally adapt an Italian version of the Communication Assessment Tool (CAT) developed by Makoul [9]. The CAT is a reliable and valid instrument for measuring patient perceptions of physician performance in the area of interpersonal and communication skills; it includes 14 items that gauge patient perceptions of physician communication, all measured on a 5-point response scale (1 = poor; 2 = fair; 3 = good; 4 = very good; 5 = excellent).

Scale development processes and psychometric properties are detailed in the original CAT article [9]. In short:

The CAT benefited from a careful review of prominent models to generate a list of communication tasks, focus groups to gather patient perspectives on items and response scales, a national survey to determine the importance attached to each item, expert review to ensure a comprehensive list of items, Lexile analysis to assess readability, and psychometric analyses to determine the most viable response scale. The plan and procedure of item generation ensured content and construct validity; scores also exhibited expected relationships with patient satisfaction data, establishing predictive validity [9].

More specifically, the 14 core items are properly considered one factor, and overall scale reliability is very high (Chronbach’s alpha = 0.96); in terms of literacy, the CAT is written at a 4^th^-grade reading level [9]. Furthermore, Differential Item Functioning analyses clearly showed that the CAT yields unbiased data for participants with different sociodemographic and clinical characteristics (i.e., items perform similarly across physician specialty as well as across patient sex, race/ethnicity, education level, self-reported health status, and previous visits to the physician [9].

The 14 core items are listed in Table 3; the Italian version of the CAT is reproduced in Additional file 1. At root, the CAT is a rating scale composed of communication tasks that are highly valued by patients and that provide actionable information for physicians (i.e., a sense of things to address do if scores are low) [9]. Taken together, the 14 CAT items are accounted for by one factor [9]. Moreover, the responses are easily quantifiable and appropriate for mathematical analysis. One potential disadvantage of rating scales is that respondent perspectives are limited to areas addressed by ratings of particular items. Accordingly, the CAT incorporates a section for comments, providing patients with an open-ended opportunity to expand on their ratings or to mention aspects not covered by the items themselves.

The instrument also includes 1 item to capture patients’ global rating of care, as well as basic demographic items. Conceptually, the CAT focuses on the achievement of communication tasks rather than prescribing particular ways of accomplishing them. At a very practical level, it is a simple and straightforward tool with discrete items that are accessible to patients across literacy levels. The CAT can be successfully completed by patients across clinical specialties, and has been used in many countries. Reporting the proportion of excellent ratings given by patients has been demonstrated to be more useful than summarizing scores via means, which are highly skewed [9].

## Methods

### Translation and cross-cultural adaptation of the CAT

**Fig. 1:**
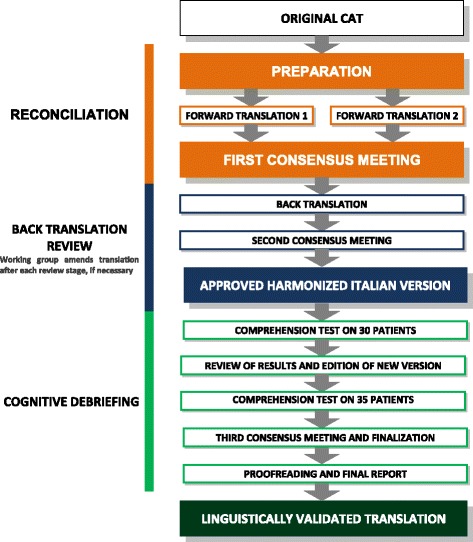
Translation and cross-cultural adaptation process flow chart

To optimize use in the Italian context, we performed translation and cross-cultural adaptation of the CAT according to internationally accepted and recommended guidelines of International Society of Pharmacoeconomics and Outcome Research (ISPOR) [10], data from international literature [11], and recommendations made by the World Health Organization (WHO) about the process of translation and adaptation of instruments [12]. The focus was on cross-cultural and conceptual equivalence, rather than simply linguistic/literal equivalence [13, 14]. The process was carried out systematically, and involved the steps illustrated in Fig. 1:

1. Preparation
2. Forward Translation
3. Reconciliation
4. Back Translation
5. Back Translation Review
6. Cognitive Debriefing
7. Review of Cognitive Debriefing Results and Finalization
8. Proofreading and Final Report

The study was performed in the out-patient clinic of the Surgical Department of Cardarelli Hospital in Naples, Italy. The study was approved by the Ethical Committee of Cardarelli Hospital and patients gave written informed consent before joining the study. A working group was set up to manage the translation and research process. It consisted of the instrument developer (GM), the chief of surgery department (MDP), three university researchers with expertise in statistic and in patient reported outcomes (VMM; VO; EM), one pharmacist with expertise in health promotion and patient education/counseling (DS), two physicians (MFA; FM) fluent in English (Cambridge certificate) with Italian as their native language, one professional translator (GT). As far as the translation is concerned, in Italy there is no professional accreditation for specialized translators. Authorized/sworn translators can be recognized on the basis of educational qualifications alone.

- ***Step 1: Preparation***. Initial work carried out before the translation work began involved obtaining permission to use the instrument, and inviting instrument developer to be involved in the study.
- ***Step 2: Forward Translation***. The translation from the English original version into Italian was carried out in parallel by two independent professional translators who are Italian native speakers with English as their first foreign language. Instructions were given to translators in the approach to translating, emphasizing conceptual rather than literal translations, as well as the need to use natural and acceptable language for the broadest audience.
- ***Step 3: Reconciliation***. The two Italian versions were compared and discussed in a consensus meeting between the two translators and the working group of the study to reach a *reconciled Italian version* (see Additional file 2).
- ***Step 4: Back Translation***. Back translation of the *reconciled Italian version* into English was carried out by a native English-speaking translator who is fluent in Italian. The English native speaker was blind to the intent and concepts underlying the material.
- ***Step 5: Back Translation Review***. In the second consensus meeting between the native English-speaking translator and the working group, the English original version was compared to the back-translated one and differences were debated, resulting in the revision, which we termed the *harmonized Italian version*.
- ***Step 6: Cognitive Debriefing***. A comprehension test for the *harmonized Italian version* was carried out in order to assess if the questionnaire was easy to understand. The questionnaire was tested on 30 patients in the out-patient clinic of Surgical Department. Over three consecutive days, all patients who visited the general or vascular surgery clinics were asked to participate into the study. Patients with cognitive deficit were excluded; the original CAT was designed to be accessible across literacy levels, but was not tested for use across different levels of cognitive function. Thirty patients out of 50 who attended the outpatient clinic agreed to participate and signed the informed consent. Respondents were administered the *harmonized Italian version* of the CAT and were systematically asked for what they thought each question was asking, whether they could repeat the question in their own words, what came to their mind when they heard a particular phrase or term. They were also asked to explain how they chose their answer.
- ***Step 7: Review of Cognitive Debriefing Results and Finalization***. Information about comprehension of items and answer mode were collected, analyzed and discussed. Test findings led to a *refined Italian version*, which was tested and validated on 35 additional out-patients, who were recruited in the same way as the sample described in Step 6. Respondents were administered the *refined Italian version* in the same way as the previous version. Suggestions and comments expressed by respondents were collected and analyzed, yielding a *final Italian version*.
- ***Step 8: Proofreading and Final Report***. The final Italian version of the CAT was reviewed carefully by the working group, and is included as Additional file 1.

### Evaluation of patient perceptions of communication with physicians engaged in the validation process

Patients involved in the translation and cultural adaptation process were asked to complete the CAT, providing a means of ensuring feasibility of using the translated and culturally adapted version in practice. We conducted a descriptive analysis of CAT scores collected during the process. As noted in the original scale development article [9], psychometric analysis indicated that “excellent” maps onto “yes”, and all of the other response options map onto “no”. Accordingly, and consistent with previous use of the CAT, results are presented as the percent of participants who gave ratings of “excellent”.

With respect to data analysis, frequencies and proportions were used to describe the characteristics of this sample as well as the CAT score for each item. The percentage of excellent responses was calculated from the total number of respondents to the individual question. Chi-squared tests were used to compare the proportion of patients who rated a given item excellent. Cronbach’s alpha internal consistency reliability was computed to assess internal consistency for the overall score of the translated CAT. Analysis were performed using SPSS Statistics for Windows, version 17.1 (SPSS Inc. Released 2008. Chicago, IL; USA).

## Results

### Translation and cultural adaptation of the CAT

We tested the Italian versions on a total of 65 patients (see Table 1 for demographics). Overall, a small number of patients had difficulty understanding the translated items (Table 2).

**Table 1:** Demographic characteristics of patients completing the CAT (*n* = 65)

	n	%
Gender		
Male	27	41,5
Female	38	58.5
Age		
24 or younger	1	1.5
25–44	22	33.8
45–64	24	36.9
65–84	18	27.7
85 or older	-	-
Nationality		
Native Italian speaker	63	96.9
Non-native Italian speaker	2	3.1
Had the patient seen this physician before?		
No	49	75.4
Yes, but only once	4	6.2
Yes, more than once	12	18.5

**Table 2:** First and second comprehension test results: percentage of the patients reporting difficulties in understanding items and questions

	First comprehension test (*n* = 30)	Second comprehension test (*n* = 35)
Items	Patients reporting difficulties in understanding items (%)	Patients reporting difficulties in understanding items (%)
4	6.7	0
9	6.7	0
15	0	5.7
	Patients reporting difficulties in understanding questions (%)	Patients reporting difficulties in understanding questions (%)
Demographic 1	30.0	0
Demographic 3	13.3	0
Demographic 4	6.7	0

The results of the first comprehension test with a group of surgical out-patients (*n* = 30) indicated that two patients, both of whom were Ukrainian, found it hard to understand items 4 and 9. Accordingly, both items were modified, since Ukrainian people are increasingly numerous in Italy. As they identified a specific word with which they were not familiar (*compreso*), we substituted a more common term (*capito*). We also re-phrased item 8, since it included a form of the same unclear word. In other words, the initial literal translation was accurate; cultural adaptation required further adjustment.

Other difficulties were not directly related to the CAT items but to demographic questions that accompany the CAT. Patients asked for clarifications about question 1, 3 and 4: question 1 investigated the age of the respondent and was organized in age categories; question 3 asked about the patient’s previous contact with the physician, and question 4 asked about respondent’s race or ethnicity. Accordingly, we modified each of these questions, taking into account suggestions expressed by participants during the interviews, which made each item more user-friendly. More specifically, we asked for age rather than age category, re-phrased the question about previous contact with the physician, and asked about “nationality” instead of race or ethnicity.

In terms of instructions, the standard instruction for patients on the questionnaire was *“please circle the chosen number”*, and 21 respondents put an “X” instead of a circle. Given this observation, we simply changed the instructions, and asked patients to indicate the chosen answer with an “X”.

The second comprehension test, with another group of surgical out-patients (*n* = 35), indicated that the adjustments were successful, as the same issues did not arise. However, two respondents were confused about the word *“your”* doctor included in item 15, which asks about the care provided by the doctor: They wondered whether *“your”* was referred to their General Practitioner or to the physician they met at that moment. We changed the item to sharpen the focus on the physician they saw during their current visit.

Results of these tests yielded an acceptable linguistic and cross-cultural adaptation of the Italian CAT version. The *final Italian version* of the CAT is in the Additional file 1.

Reliability was tested through the analysis of internal consistency. Results indicate that the overall scale reliability is very high for the 14-items of Italian CAT version (Cronbach’s alpha = 0.95).

### Evaluation of patients’ perceptions of communication with the physicians engaged in the validation process

The 65 patients who completed the CAT had a broad age distribution and were predominantly female (58.5 %). There were no significant differences in the overall percentage of items rated as excellent when comparing results based on patient age or gender. The scores on individual CAT items ranged from 36.9 to 69.2 % excellent (Table 3). The highest-scoring items were “Talked in terms I could understand” at 69.2 % and “Paid attention to me (looked at me, listened carefully)” at 64.6 %. The lowest-scoring item was “Encouraged me to ask questions” at 36.9 %, a finding that matches other published studies using the CAT [9, 15–20]. These results are a sign that the instrument is operating as expected. Given the sample size and purpose (i.e., refining the translation and cultural adaptation), they should not be considered representative of how patients view communication with surgeons in Italy.

**Table 3:** Percentage of excellent ratings for individual CAT items

CAT item	Ratings (% Excellent) (*N* = 65)
1. Greeted me in a way that made me feel comfortable	56.9
2. Treated me with respect	63.1
3. Showed interest in my ideas about my health	49.2
4. Understood my main health concerns	50.8
5. Paid attention to me (looked at me, listened carefully)	64.6
6. Let me talk without interruptions	61.5
7. Gave me as much information as I wanted	63.1
8. Talked in terms I could understand	69.2
9. Checked to be sure I understood everything	58.5
10. Encouraged me to ask questions	36.9
11. Involved me in decisions as much as I wanted	55.4
12. Discussed next steps, including any follow-up plans	61.5
13. Showed care and concern	63.1
14. Spent the right amount of time with me	60.0

## Discussion

To our knowledge, this is the first study conducted in Italy on translation and cultural adaptation of any instrument assessing patients’ perception of physicians’ ability to communicate with them. Effective communication is integral to high-quality care and has been linked to improved patient outcomes and compliance with physician recommendations. Several national organizations have recognized the importance of communication between physicians and patients. For instance, the Italian Health Ministry has developed several projects to improve communication within clinical settings. Moreover, an increasing number of healthcare organizations use patient satisfaction ratings, including satisfaction with physicians’ communication skills, when determining physician compensation and for referring physicians whose skills are deficient to specialized educational programs [21, 22]. In this study, the well validated and highly reliable CAT [9] was successfully translated and cross-culturally adapted to Italian context. The process was carried out according to standardized procedures and after a multi-step process with corrections and adjustments taking into account not only linguistic factors but also cultural components [10–12]. The use of detailed methods was essential to document development of an equivalent version of the CAT that is appropriate for use in Italy.

The CAT is a feasible tool to assess patient perceptions of physician communication, and offers a rare but essential opportunity for providing physicians with systematic feedback. The translation and cross-cultural adaptation of an instrument to assess interpersonal and communication skills has useful implications for practice since there are currently no validated clinical scales in the Italian language. The CAT can help physicians reflect on their interpersonal and communication skills, with the goal of reinforcing strengths and identifying areas that deserve more attention for improvement. It may have also useful implications for collecting information on physicians’ communication needs in order to plan tailored training programs. This study represents the first step in the translation and cultural adaptation of the CAT instrument in Italy. The second step is to validate the instrument’s psychometric properties in larger Italian samples. The same research team is working to involve surgical departments across Italy to reach a much larger sample of patients. The results will consolidate and validate the instrument for the Italian population, while providing a more representative view of how patients view communication with surgeons in Italy.

The analysis of patient perceptions of communication with the physicians found patterns similar to those shown in previous studies using the CAT [9, 15–18]. In particular, patients desire more opportunities to ask questions and more active involvement in decisions regarding their care as well as more of a sense that their physicians are interested in their ideas about their own health and their health concerns, reinforcing the importance of patients’ ability to choose among possible solutions (choice right) and take more responsibility for their care (patient empowerment).

Communication skills can effectively be taught, and continuing medical education programs could incorporate these skills. Building on excellent programs for teaching new surgical skills, surgeons could be taught how to discuss surgical procedures with simulated patients, which would include exploring fears associated with particular procedures and learning to discuss these issues in an effective, efficient manner. A critical component would be to incorporate more robust communication skill training into health professionals’ university curricula.

As reported in literature, Dr. Francis Peabody of the Harvard Medical School realized the need for these qualities in the 1920s, stating “young graduates have been taught a great deal about the mechanism of disease, but very little about the practice of medicine or, to put it more bluntly, they are too ‘scientific’ and do not know how to take care of patients” [23]. Another author with similar viewpoints believed a clinician’s armamentarium consisted of “the herb, the knife and the word” [24]. Healthcare education is greatly devoted towards both ‘the herb’ and ‘the knife,’ while ‘the word’ is oftentimes forgotten or pushed aside in the education of health care professionals. However, this does not make ‘the word’ any less important.

## Limitations

A limitation of the study is that we used the Italian version of the CAT only in surgical patients and not in other specialties. We choose the Surgical Department because Italian Scientific Surgeon Associations are showing an increasing interest in this field and little has been done on this topic until recently. Previously published studies have shown that CAT items transcend specialty, although scores differ across specialties [9, 15–17].

## Conclusions

In summary, we have provided Italian clinical practice with a robust instrument for assessing patient perception of physicians’ communication abilities. This adaptation exhibited a satisfactory level of semantic equivalence between the Italian target and the original English source version. The Italian version was tested in the surgical out-patient setting, but is expected to be equally viable in other settings such as Primary Care and Pharmacy where effective interpersonal and communication skills are particularly crucial keys to improving clinical outcomes and treatment adherence, especially in chronic diseases.

## Availability of data and materials

The dataset supporting the conclusions of this article is included within the article as Additional file 3.

## Additional files

Additional file 1: Final version of the CAT translated and culturally adapted for Italian clinical practice. (PDF 152 kb) Additional file 2: Table S1. Short description of the modifications made to the *reconciled Italian version*, *Harmonized Italian version*, and *Refined Italian version*. (DOCX 14 kb) Additional file 3: Dataset. (XLSX 15 kb)

## Abbreviations

CAT: communication assessment tool
WHO: World Health Organization
