# Supplementary material for: Italian translation and cultural adaptation of the communication assessment tool in an outpatient surgical clinic
Source: BMC Health Serv Res. 2016 Apr 29;16:163. doi: 10.1186/s12913-016-1411-9 (PMC4851771; doi:10.1186/s12913-016-1411-9)
Supplement: Additional file 1: — Final version of the CAT translated and culturally adapted for Italian clinical practice. (PDF 152 kb) [file 12913_2016_1411_MOESM1_ESM.pdf]

Appendix
Ownerships: the questionnaire as well as its translation, adaptations, computer programs, and scoring of the CAT are intellectual property of Gregory Makoul, Gregory Makoul, PhD, MS
Director, Connecticut Institute for Primary Care Innovation, Hartford, CT, USA
Professor of Medicine, University of Connecticut School of Medicine, Farmington, CT, USA and can be obtained through a license agreement with the Developer/Owner

**Nome del Medico:**

Strumento di Valutazione della Comunicazione

La comunicazione con i pazienti è una componente molto importante della qualità dell'assistenza medica. Gradiremmo conoscere le sue impressioni sul modo con cui il suo medico comunica con lei. **Le sue risposte sono del tutto confidenziali, per cui Le saremo grati se sarà il più possibile sincero ed obiettivo**.
La sua partecipazione è volontaria e non influirà in alcun modo sull'assistenza medica.

Per favore dia un punteggio al modo di comunicare del suo medico.
Segni con una X la sua risposta per ciascuna domanda mostrata di seguito.
Grazie molto

**Il Medico…**	**Scarso**	**Sufficiente**	**Buono**	**Molto Buono**	**Eccellente**
1. Mi ha accolto in un modo che mi ha fatto sentire a mio agio	1	2	3	4	5
2. Mi ha trattato con rispetto	1	2	3	4	5
3. Ha mostrato interesse per le mie idee sulla mia salute	1	2	3	4	5
4. Ha capito le mie principali preoccupazioni di salute	1	2	3	4	5
5. Mi ha prestato attenzione (mi ha guardato, mi ha ascoltato con attenzione)	1	2	3	4	5
6. Mi ha lasciato parlare senza interrompermi	1	2	3	4	5
7. Mi ha fornito tutte le informazioni che volevo	1	2	3	4	5
8. Ha parlato con parole per me facili da capire	1	2	3	4	5
9. Ha verificato che avessi capito ogni cosa	1	2	3	4	5
10. Mi ha incoraggiato a fare domande	1	2	3	4	5
11. Mi ha coinvolto nelle decisioni sulla mia salute nella misura da me desiderata	1	2	3	4	5
12. Ha discusso sulle prossime cose da fare, incluso eventuali programmi di esami e visite di controllo	1	2	3	4	5
13. Ha mostrato attenzione e interesse	1	2	3	4	5
14. Mi ha dedicato il giusto tempo	1	2	3	4	5

~ continua sull'altro lato ~

Appendix

Ownerships: the questionnaire as well as its translation, adaptations, computer programs, and scoring of the CAT are intellectual property of Gregory Makoul, Gregory Makoul, PhD, MS
Director, Connecticut Institute for Primary Care Innovation, Hartford, CT, USA
Professor of Medicine, University of Connecticut School of Medicine, Farmington, CT, USA and can be obtained through a license agreement with the Developer/Owner

Copyright © 2004/2010 – Gregory Makoul, PhD – All rights reserved – Non-commercial, educational use permitted

**Le sue cure**	**Scarso**	**Sufficiente**	**Buono**	**Molto Buono**	**Eccellente**
15. Quale punteggio darebbe all'assistenza fornita da questo medico?	1	2	3	4	5

Commenti:

****

Queste domande servono per scopi statistici. Le sue risposte rimarranno anonime.
Per favore segni una sola risposta per ogni domanda.

1. Quale è la sua età? ____

2. Quale è il suo sesso?
   - ☐1 Maschio
   - ☐2 Femmina

3. Ha mai avuto contatti con questo medico prima?
   - ☐1 No
   - ☐2 Si , ma solo una volta
   - ☐3 Si, più di una volta

4. Nazionalità:
   - ☐1 Italiana
   - ☐2 Non Italiana

5. Oggi era lei il paziente?
   - ☐1 Si
   - ☐2 No, ho accompagnato il paziente

Grazie molto
