# Supplementary material for: Italian translation and cultural adaptation of the communication assessment tool in an outpatient surgical clinic
Source: BMC Health Serv Res. 2016 Apr 29;16:163. doi: 10.1186/s12913-016-1411-9 (PMC4851771; doi:10.1186/s12913-016-1411-9)
Supplement: Additional file 2: Table S1. — Short description of the modifications made to the reconciled Italian version, Harmonized Italian version, and Refined Italian version. (DOCX 14 kb) [file 12913_2016_1411_MOESM2_ESM.docx]

| Section/Item | Reconciled Italian Version | | Back Translation Review | | Harmonized Italian Version | | Refined Italian Version | | Final Italian version | |  |
| --- | --- | --- | --- | --- | --- | --- | --- | --- | --- | --- | --- |
| Title | Discussion | Consensus | Discussion | Consensus | Discussion | Consensus | Discussion | Consensus | Discussion | Consensus |  |
| Instruction |  |  |  |  | Segni con un cerchio la sua risposta per ciascuna domanda mostrata di seguito | *Changed to*  Segni con una X la sua risposta per ciascuna domanda mostrata di seguito |  |  |  |  |  |
| CAT  Item 1 | Mi ha accolto in un modo che mi ha fatto sentire a proprio agio  Mi ha salutato in modo accogliente | *Changed to*  Mi ha accolto in un modo che mi ha fatto sentire a mio agio |  |  |  |  |  |  |  |  |  |
| CAT  Item 3 |  |  | Si è interessato alle mie idee sulla mia salute | *Changed to*  Ha mostrato interesse per le mie idee sulla mia salute |  |  |  |  |  |  |  |
| CAT  Item 4 |  |  |  |  | Ha compreso le mie principali preoccupazioni di salute | *Changed to*  Ha capito le mie principali preoccupazioni di salute |  |  |  |  |  |
| CAT  Item 7 |  |  |  |  |  |  |  |  | Mi ha fornito le informazioni di cui avevo bisogno | *Changed to*  Mi ha fornito tutte le informazioni che volevo |  |
| CAT  Item 8 |  |  |  |  | Ha parlato con parole per me comprensibili | *Changed to*  Ha parlato con parole per me facili da capire |  |  |  |  |  |
| CAT  Item 9 |  |  |  |  | Ha verificato che avessi compreso ogni cosa | *Changed to*  Ha verificato che avessi capito ogni cosa |  |  |  |  |  |
| CAT  Item 11 | Mi ha coinvolto nelle decisioni tanto quanto volevo  Mi ha coinvolto nelle decisioni nella misura da me desiderata | *Changed to*  Mi ha coinvolto nelle decisioni sulla mia salute nella misura da me desiderata |  |  |  |  |  |  |  |  |  |
| CAT  Item 15 | | Che punteggio Lei dà all’attenzione fornita dal Suo medico?  Quale punteggio darebbe alle cure fornite dal suo medico? | *Changed to*  Quale punteggio darebbe all’assistenza fornita dal suo medico? |  |  |  |  | Quale punteggio darebbe all’assistenza fornita dal suo medico? | *Changed to*  Quale punteggio darebbe all’assistenza fornita da questo medico? |  |  |
| Demographic  Question 1 | |  |  |  |  | Age categories | *Changed to*  Patient’s age |  |  |  |  |
| Demographic  Question 3 | |  |  |  |  | Ha mai incontrato questo medico prima? | *Changed to*  Ha mai avuto contatti con questo medico prima? |  |  |  |  |
| Demographic  Question 4 | |  |  |  |  | Race and ethnicity | *Changed to*  Nationality |  |  |  |  |
